# Emerging Arboviral Diseases in Pakistan: Epidemiology and Public Health Implications

**DOI:** 10.3390/v17020232

**Published:** 2025-02-07

**Authors:** Muhammad Ammar, Muhammad Moaaz, Chaoxiong Yue, Yaohui Fang, Yanfang Zhang, Shu Shen, Fei Deng

**Affiliations:** 1 Key Laboratory of Virology and Biosafety and National Virus Resource Center, Wuhan Institute of Virology, Chinese Academy of Sciences, Wuhan 430071, China; ammar.sz@hotmail.com (M.A.); ycx237614999@outlook.com (C.Y.); fyh@wh.iov.cn (Y.F.); zhangyf@wh.iov.cn (Y.Z.); 2 University of Chinese Academy of Sciences, Beijing 100049, China; 3 KBCMA, College of Veterinary and Animal Sciences, Narowal, University of Veterinary and Animal Sciences (Sub-Campus), Lahore 54000, Pakistan; muhammadmoaaz2003@gmail.com

**Keywords:** arboviruses, mosquito-borne diseases, tick-borne diseases, epidemiology, public health, Pakistan

## Abstract

Arboviruses pose significant public health challenges globally, particularly in Pakistan, where deforestation, climate change, urbanization, inadequate sanitation, and natural disasters have all contributed to the spread of mosquito-borne flavivirus diseases like dengue fever. The lack of a thorough national surveillance system has made it difficult to determine the extent and distribution of these diseases. Concern has been raised by recent outbreaks of West Nile virus (WNV) and chikungunya (CHIKV) epidemics, which may lead to Zika virus (ZIKV) outbreaks in the future. Additionally, hospital-based surveillance has detected the Japanese encephalitis virus (JEV) in the region. Evidence also points to the presence of additional arboviruses in healthy populations, such as the Karshi virus (KSV), Tamdy virus (TAMV), Crimean–Congo hemorrhagic fever virus (CCHFV), and severe fever with thrombocytopenia syndrome virus (SFTSV). This review aims to address the risk factors linked to these diseases, provide specific policy recommendations for efficient disease prevention and control, and describe the epidemiological trends of these diseases in Pakistan while emphasizing the critical need for improved surveillance and thorough epidemiological investigations.

## 1. Introduction

**Figure 1:**
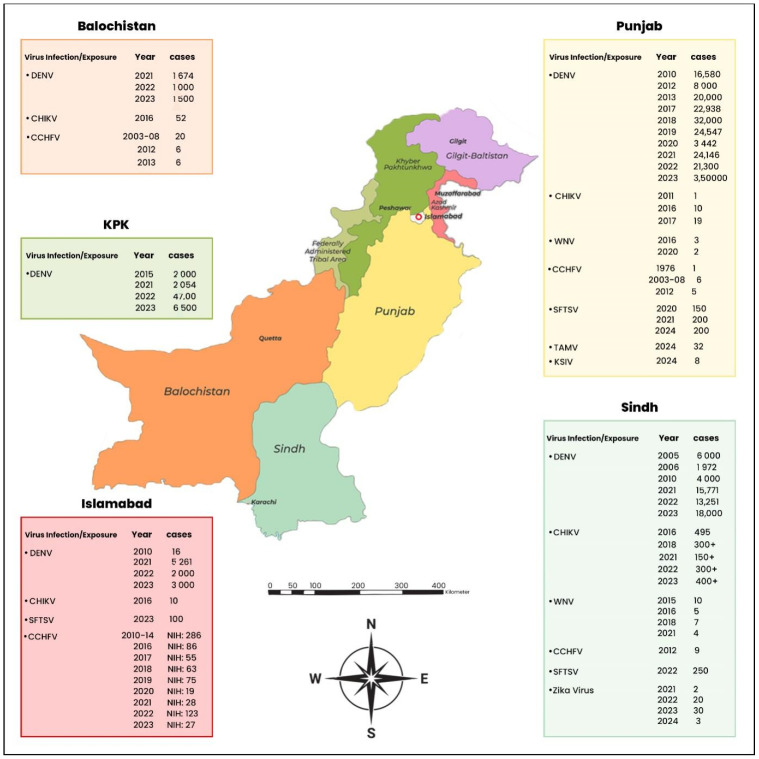
Annual distribution of reported arbovirus cases across provinces in Pakistan, highlighting regional incidence variations.

Pakistan, which occupies an ideal location at the meeting point of south, central, and west Asia, has several different climates and ecosystems that contribute to the development of different mosquito-borne diseases (MBDs), which presents serious public health issues. Approximately two-thirds population of country lives in rural regions, with livelihoods that rely on agricultural and animal husbandry. This increases their exposure to livestock and wildlife, which may play a role in the transmission of zoonotic infections, including arboviruses [1,2]. Therefore, vector-borne illnesses including chikungunya, dengue, and Crimean–Congo hemorrhagic fever (CCHF) continue to pose a serious threat to public health. The dengue virus (DENV) has become more common since the first dengue fever case was discovered in Pakistan in 1994; cases have been documented in all five provinces [3]. Following this, chikungunya, which is spread by *Aedes mosquitoes* like dengue, appeared in 2016–2017 [4]. Additionally, CCHFV, which was initially discovered in the area in 1976, is still a major problem as cases are still being recorded [5,6]. Moreover, the rise of other arboviral infections like the Tamdy virus (TAMV), Zika virus (ZIKV), and severe fever with thrombocytopenia syndrome virus (SFTSV) are posing an alarming situation for public health officials of Pakistan. Furthermore, the concerning CCHFV seroprevalence of 7.59% in the same area highlights the country’s growing tick-borne virus burden. To reduce the hazards posed by these newly emerging viruses, these findings necessitate increased surveillance and public health initiatives (Figure 1) [7].

There are several reasons that these diseases are spreading more widely. A major factor is climate change since seasonal monsoon rainfall and warming temperatures provide ideal circumstances for mosquito vector growth. According to research, the probability of disease transmission is increased by temperatures between 26 °C and 29 °C, which are especially favorable for vector growth [8]. Research conducted in Lahore shows that mosquito populations drastically decrease when temperatures drop below 16 °C, as tropical mosquitoes can successfully finish their life cycles at higher temperatures [9]. The epidemic of dengue and other infections was made worse by the catastrophic flooding events that occurred in 2010–2012, which increased mosquito exposure. During that time, Pakistan recorded the largest number of dengue cases in decades [10]. Rapid economic development and urbanization have contributed to the spread of diseases carried by mosquitoes. *A. aegypti* and *A. albopictus*, the main vectors of dengue and chikungunya, flourish in overcrowded cities with inadequate infrastructure [11]. The danger of outbreaks is further increased by the poor management of water and waste resources in urban slums, which makes it more likely for mosquitoes to spawn in standing water. The spread of these diseases has also been aided by globalization, which has led to an increase in trade and travel. For example, *A. aegypti*, originally native to Africa, has spread to other parts of the world including Pakistan, primarily due to international trade and the movement of used tires, which serve as a significant mosquito dispersal [12]. The potential of arboviral infection transmission across borders is further increased by Pakistan’s main cargo port, which is situated on the Arabian Sea [13].

The public health hazards linked to diseases conveyed by mosquitoes are made worse by the country’s pervasive unsanitary conditions. There exists an abundance of water supply alongside a lack of clean drinking water, improper sanitation, and ineffective waste management practices in Pakistan, which facilitates the breeding of mosquito larvae. Due to these factors, vector-borne diseases like dengue and chikungunya have become endemic, particularly in slum communities in both rural and urban locations [14].

Furthermore, the observed circulation of neutralizing antibodies in seroprevalence study-positive samples highlights ongoing infection routes for viruses carried by ticks and mosquitoes. To address the threats posed by these arboviruses and their vectors, awareness campaigns and preventive measures are needed [7,15,16,17]. There is an urgent need for improved surveillance, focused research, and all-encompassing public health interventions due to the increasing prevalence of diseases carried by mosquitoes and ticks, as well as the ecological and behavioral variables that contribute to their proliferation. The objective of this review is to present a comprehensive overview of the epidemiology of these vector-borne viral infections in Pakistan, with an emphasis on pathogens that are spread by ticks and mosquitoes.

## 2. Epidemiological Trends of Arboviral Diseases in Pakistan: Mosquito-Borne and Tick-Borne Infections

### 2.1. Epidemiological Trends of Mosquito-Borne Diseases in Pakistan

#### 2.1.1. Dengue Fever

Since the first recorded outbreak of dengue fever in 1994, involving over 1000 cases in Karachi, the epidemiological landscape of the disease in Pakistan has significantly changed [18]. Dengue has become more widespread and contagious, especially due to urbanization and environmental changes [19].

Dengue fever has evolved from a rare occurrence to a widespread health risk in Pakistan. Dengue expanded to neighboring provinces during an epidemic in 2005, when 395 cases were confirmed in Karachi, thereby becoming endemic across the country [19,20,21]. According to data from the National Institutes of Health, the number of cases has been on the rise, with 22,938 cases recorded in 2017 and 48,906 cases in 2021 [2]. This increasing trend continued as of October 2022, when 41,746 confirmed dengue cases were reported, mostly in Sindh province as a result of heavy flooding (Figure 1, Table 1) [2].

One of the main causes of the increase in dengue cases is climate change. Around 33 million people were affected by the historic flooding in mid-2022, according to Pakistan’s climate change minister, which raised the risk of mosquito-borne diseases even further [20]. The spread of dengue has been facilitated by a confluence of unsanitary conditions, stagnant water, and inappropriate urban design. The collapse of drainage systems has caused problems in Karachi in particular, flooding neighborhoods with stagnant water mixed with sewage and creating breeding grounds for mosquitoes such as *A. aegypti* and *A. albopictus* [20]. The World Health Organization (WHO) has also observed that the prevalence of dengue is made worse by favorable monsoon weather and insufficient vector control methods. The effects of climate change, particularly in the form of excessive rainfall, highlight the connection between environmental factors and the breakdown of health infrastructure [22,23]. The current situation is made more complex by the prevalence of dengue serotypes. Effective surveillance and prompt response are essential because the coexistence of all four serotypes in Pakistan increases the risk of severe disease through re-infection [24,25]. Pakistan announced 2795 new dengue cases by September 2024, primarily from Balochistan, indicating a serious public health crisis that puts a burden on healthcare systems across the country [25].

Over 1000 dengue patients might need to be treated simultaneously in towns like Rawalpindi, putting significant strain on healthcare infrastructure. This burden emphasizes the necessity of an integrated strategy that addresses the socioeconomic factors influencing the spread of disease as well as preventive and therapeutic interventions [26].

To reduce the dengue outbreak, a multisectoral strategy is necessary. It is essential to upgrade infrastructure, especially drainage systems, to avoid standing water buildup, which provides mosquitoes with a breeding site. Eliminating *Aedes* mosquito habitats in urban settings requires effective water management techniques [27]. Furthermore, improving health surveillance systems, educating healthcare professionals, and guaranteeing prompt case management are all essential components of better epidemic control. The national dengue response plan can be improved through cooperation with foreign partners by utilizing their resources and knowledge [28]. Establishing long-term solutions requires investing in creative mosquito control techniques in addition to prioritizing sustainable vector control measures. To obtain funds for epidemic preparedness and to support public health initiatives, it is also essential to advocate for resource mobilization on a national and international scale [29].

#### 2.1.2. Chikungunya Virus

The chikungunya virus (CHIKV), an arbovirus which spread primarily by *Aedes mosquitoes*, has reappeared as a significant public health problem in Pakistan. The virus was first discovered in the country in 1983, but it paused before making a comeback during a significant epidemic that began in December 2016 [30]. The CHIKV virus was first discovered in Pakistan in 1983, but it was not reported until the 2016 outbreak that it made a significant comeback [30]. According to the Ministry of Health Services, Regulation, and Coordination (NHSRC), the WHO formally recognized the outbreak in December 2016 and found that over 30,000 suspected cases had been confirmed by RT-PCR [31]. Health officials observed that the first wave of infections occurred during a time when temperatures rose, creating ideal circumstances for mosquito breeding [32]. The virus has thrived in the country due to its geographic and climatic characteristics, which have been exacerbated by urbanization. The problem has been made worse by rapid population expansion and poor sanitary infrastructure, which have given mosquitoes perfect breeding grounds and caused catastrophic epidemics in big cities like Karachi (Figure 1, Table 1) [31,33].

One important aspect affecting Pakistan’s chikungunya epidemiology is climate change. According to reports, the rise of arboviral diseases like chikungunya and other diseases like dengue has been facilitated by rising temperatures and unpredictable rainfall patterns [31]. This is made worse by the poor hygienic conditions that are common in many metropolitan areas, especially in Karachi and other cities. The absence of effective vector control methods exacerbates the public health emergency, underscoring the need for prompt actions, including targeted strategies for vector reduction and improvements in sanitary infrastructure to prevent further transmission [30]. Although chikungunya can cause a variety of symptoms, its main ones are rash, severe joint pain, and a high fever. Following an acute infection, patients may occasionally experience severe aftereffects that include neurological, cardiovascular, pulmonary, renal, ophthalmic, and cutaneous issues [31]. The crippling joint pain is frequently chronic, greatly affecting quality of life and adding to the strain on medical services. The reappearance of chikungunya is a significant public health challenge for Pakistan, especially given the demand for already scarce healthcare resources caused by concurrent outbreaks of other mosquito-borne diseases like dengue. Reducing the danger of CHIKV and other arboviral infections requires efficient management, surveillance, and public health education programs [34]. A diversified strategy is required to effectively handle the chikungunya epidemic. To reduce the spread of viruses by mosquitoes, it is essential to implement both preventative measures, such as eliminating standing water, and control strategies, such as the use of insecticides [35,36]. Improving urban sanitation and drainage infrastructure is essential to reducing mosquito breeding grounds. Resolving these infrastructure issues will significantly reduce the likelihood of future outbreaks [37]. Strong surveillance measures must be put in place to identify CHIKV patients early. Early detection of outbreaks can help with quick response times and reduce the virus’s ability to spread throughout populations [38,39]. Further investigation of the pathogenicity and epidemiology of CHIKV will contribute to the development of public health initiatives. Working together with global health organizations can improve resource mobilization and strengthen outbreak response [39,40].

#### 2.1.3. West Nile Virus (WNV)

The arbovirus known as West Nile virus (WNV), which is mainly spread by mosquitoes of the Culex genus, has become a serious public health issue in Pakistan ever since it was first isolated in 1966 [41]. The virus has become established in the ecosystems of the country, and both domestic and wild animal populations are affected [42]. The purpose of this review is to look at the epidemiological patterns of West Nile virus (WNV) in Pakistan, identify challenges in its surveillance and management, and discuss strategies for improving prevention and control. Significant rates of WNV prevalence were found in a variety of animal species in later research, most notably a seroepidemiological evaluation carried out in Punjab Province. West Nile virus (WNV) primarily depends on birds as its main reservoirs. Domestic animals, such as dogs and horses, and wild animals, including squirrels, blue jays, and crows, can also act as important reservoirs in the transmission cycle. The need for better surveillance techniques has been highlighted by reports from more recent years that show WNV infections in people can cause serious neurological disorders [17]. Effective procedures must be put in place to monitor the spread of WNV because it is present in a variety of environments.

Clinical manifestations of WNV infection in humans can vary from mild cases to serious neurological conditions including encephalitis and meningitis. In 2018, a significant serological survey found alarming evidence of neurological problems linked to WNV in humans (Table 1) [17]. The severity and symptoms of WNV infections pose a significant public health risk, inciting health authorities to improve their surveillance and early detection efforts. An urgent call to action for improved public health response mechanisms is necessary since cases of neurological diseases linked to WNV have put increasing strain on healthcare services. The severity of the situation and the necessity of prompt care are highlighted by the rising frequency of neurological symptoms linked to WNV infections [43]. Urbanization and climate change are two ecological and environmental elements that are intimately related to WNV epidemiology. The dynamics of virus transmission are significantly influenced by the geographic distribution of vector mosquitoes, especially the Culex species that are known to transmit WNV. Furthermore, Pakistan’s climate, which is marked by temperature variations and monsoon rains, might affect mosquito populations and activity levels. Developing successful control methods requires an understanding of the interactions between these environmental factors and WNV epidemiology [44]. A diversified strategy is necessary to lessen the negative effects of WNV on public health in Pakistan. The prompt detection of WNV cases and outbreaks will be made possible by the establishment of extensive surveillance systems for populations, domestic animals, and wild populations [44]. Research on the dynamics of WNV transmission and its effects on public health must continue. Improving reaction capacities and enabling prompt case management are two benefits of teaching medical personnel to identify and treat WNV infections [45].

#### 2.1.4. Japanese Encephalitis (JE)

In Pakistan, Japanese encephalitis (JE) is a serious public health concern because of the environmental factors and appropriate vectors that allow the Japanese encephalitis virus (JEV) to spread [46]. Although JEV has historically gone mostly undetected, recent serological tests show that the disease is present in the area, requiring significant advancements in monitoring and diagnostic capacities. In Asia, Japanese encephalitis is the most common viral encephalitis caused by the JEV, which is mostly spread by *Culex species*, especially *tritaeniorhynchus*. Early in the 20th century, JEV was first identified; the first cases were reported in Japan in the 1920s, and outbreaks later occurred in other Asian countries, including Pakistan. Studies conducted in Karachi throughout the 1980s and 1990s found the first recorded case of JE in Pakistan by the use of nucleic acid testing in encephalitis patients [47]. Research has verified the presence of JEV in both humans and domestic animals, underscoring the possibility of human-to-human transmission in Pakistan.

The paucity of historical JE case reporting in Pakistan has made it difficult to comprehend the effect and spread of the disease. However, the frequency of the virus is starting to become clearer through recent serological surveys. In Punjab, for instance, a seroepidemiological investigation showed high infection rates in both domestic and wild animal populations, suggesting that the virus is still spreading. In southern Punjab, immunoglobulin M (IgM) against JEV was found in individuals with feverish illnesses in 2018 [2,44]. Additionally, a study conducted between 2015 and 2018 in two acute care hospitals in Karachi confirmed six positive cases of JEV-IgM in serum and cerebrospinal fluid samples from patients diagnosed with encephalitis (Table 1) [48]. This emphasizes the urgent need for increased knowledge and enhanced diagnostic skills to properly identify and handle instances.

The clinical manifestations of JE may differ from asymptomatic infections to severe neurological disease, with a case fatality rate reported between 20% and 30%. Survivors frequently experience serious aftereffects, such as mental health issues and neurological damage. Although the majority of human JEV infections are asymptomatic, they can cause anything from a low-grade fever to potentially fatal encephalitis; roughly one in every 300 cases can result in serious complications such as meningoencephalitis or flaccid paralysis [13]. In endemic locations, the WHO estimates that the annual incidence of JE is 10% [49]. Because co-circulating flaviviruses like West Nile virus (WNV) and dengue virus (DENV) make serological testing more difficult, public health authorities in Pakistan have a difficult time detecting JE. Additionally, the Karachi investigation conducted from 2015 to 2018 revealed serological cross-reactivity with DENV and WNV, which makes it difficult to confirm or rule out local JEV transmission [49]. It is critical to increase knowledge of the risk factors for JE and the value of vaccination. The foundation for JE control is vaccination. To reduce the disease burden in high-risk locations, the JE vaccine must be incorporated into national immunization programs. Understanding the environmental factors that encourage mosquito reproduction might help communities take proactive measures to reduce hazards [16,50].

#### 2.1.5. Zika Virus (ZIKV)

ZIKV is a newly discovered mosquito-borne virus that is similar to dengue virus (DENV) and chikungunya virus (CHIKV) in terms of clinical signs and transmission vectors [51]. Nowadays, *A. aegypti* and *A. albopictus*, the main vectors of these viruses, are found in many cities in Pakistan. These mosquito species persist in their success even after control measures have been put in place due to the availability of breeding grounds [52].

Apart from its clinical resemblance to CHIKV and DENV, ZIKV is specifically linked to serious consequences such as adult Guillain–Barré syndrome (GBS) and neonatal microcephaly [53]. Though no confirmed ZIKV cases have been documented, unknown causes of newborn microcephaly and GBS have been described in Pakistan [17]. According to historical records, ZIKV was circulating in the area as early as 1983, when serological investigations found ZIKV antibodies in domestic animals, rodents, and humans [17,54]. Seroprevalence investigations conducted more recently have shown that human exposure rates of 6.48% highlight the historical presence of ZIKV and its possible effects on the Pakistani population [55].

Lack of knowledge and diagnostic ability is a significant barrier to ZIKV identification in Pakistan. Over 60% of the population lives in rural areas, where people tend to delay getting medical attention for minor ailments including rash, fever, conjunctivitis, and joint discomfort. Medical professionals are also more inclined to diagnose dengue due to its endemicity over the last 10–12 years [53]. Additionally, successful screening attempts are hampered by the lack of ZIKV-specific diagnostic kits at the local practitioner level. Laboratory confirmation of ZIKV infections is made more difficult by cross-reactivity with DENV and other flaviviruses [54].

The fact that Pakistan is geographically close to countries where ZIKV cases have been documented makes the threat of ZIKV much more serious (Table 1). In May 2016, for example, India’s health ministry confirmed three cases in Gujarat, a state that borders Sindh province in Pakistan [56]. In the same way, a ZIKV imported case was reported in February 2017 in Henan Province of China. The patient had just returned from an endemic region of ZIKV in Central America [56]. The possibility of ZIKV emergence in Pakistan is highlighted by these regional reports and the history of dengue outbreaks in the country [54].

ZIKV is a serious threat to Pakistan, according to predictive models, especially in heavily populated cities like Karachi and Lahore. Rapid urbanization, the growth of slum areas, and the rise in vector populations make these cities prime locations for a possible outbreak [15,57]. Pakistan was one of 12 Asian countries that the Centers for Disease Control and Prevention (CDC) designated as having a high risk of suffering from a ZIKV outbreak in September 2016. It is also possible that ZIKV was introduced into Pakistan by foreign visitors, according to the CDC’s qualitative analysis of inbound aviation traffic from ZIKV-endemic areas, such as Miami, Singapore, Florida, and Brazil [8,54]. It is essential to improve Pakistan’s surveillance and diagnosis capabilities because of the serious health consequences and explosive breakout potential of ZIKV.

### 2.2. Epidemiological Trends of Tick-Borne Diseases in Pakistan

#### 2.2.1. Severe Fever with Thrombocytopenia Syndrome Virus (SFTSV)

A new tick-borne virus known as severe fever with thrombocytopenia syndrome virus (SFTSV) is associated with severe hemorrhagic fever, which presents serious public health issues. According to recent seroprevalence investigations conducted in Faisalabad, Pakistan, the estimated exposure rate to SFTSV among healthy individuals is 17.38%, which is alarming [58]. Since it was first identified during an outbreak in China in 2009, SFTSV has been acknowledged as a public health concern in several East Asian countries, such as Japan and South Korea. Ticks are the main vectors of SFTSV, especially those of the genus *Haemaphysalis*, with *Haemaphysalis longicornis* being the most prevalent vector [59]. People in endemic areas are highly susceptible to infection, especially those who work in agriculture or outdoor activities, as the virus is spread to humans through tick bites.

The significance of comprehending both the spatial distribution of vectors and the dynamics of SFTSV transmission is highlighted by the seroprevalence numbers from Faisalabad. The reported exposure rate of 17.38% among healthy adults indicates that the virus is widely circulated throughout the population. Furthermore, the finding of neutralizing antibodies in a portion of positive samples raises the possibility that some people may have contracted the virus and established an immunological response. This suggests the existence of ongoing infection routes, which need careful observation [58].

Mortality rates can rise sharply as the illness frequently develops into more severe diseases such as hemorrhagic symptoms and multi-organ failure [60]. The potential severity of SFTSV infections has been highlighted by the WHO’s report of varying case fatality rates in various geographic regions, which range from 5% to over 30% [61]. The clinical symptoms of SFTSV patients vary, usually starting as mild flu-like symptoms and developing into more severe ones as the disease worsens. When the disease is advanced, infected patients may first exhibit mild symptoms like fever, exhaustion, and gastrointestinal issues before developing more serious symptoms including severe hemorrhagic signs and neurological symptoms [60,62]. Public health authorities must put in place thorough plans for tracking and managing this viral infection because of the high seroprevalence of SFTSV in Faisalabad and the health hazards it poses [63]. The establishment of improved surveillance methods is necessary to track SFTSV activity in human cases as well as among tick populations. This will allow for the prompt detection of epidemics and the application of control measures [63,64]. To guarantee prompt detection and treatment of SFTSV infections, accessible healthcare services must be improved [65]. It is critical to do ongoing research on the epidemiology, clinical features, and efficacious vaccination or treatment approaches of SFTSV. To inform focused actions, this study should concentrate on comprehending the mechanisms of transmission in local contexts (Table 1) [66].

#### 2.2.2. Crimean–Congo Hemorrhagic Fever Virus (CCHFV): An Emerging Health Threat in Pakistan

Pakistan faces a serious public health threat from the Crimean–Congo hemorrhagic fever virus (CCHFV), particularly during cultural celebrations such as Eid-ul-Azha, a major Islamic festival where the slaughtering of animals may lead to increased contact with livestock, a major source of CCHFV transmission. Tick bites, especially from members of the *Hyalomma* genus, and contact with the body fluids of infected livestock are the main ways by which this arbovirus is spread. According to recent studies, the population in Faisalabad has an alarming seroprevalence of 7.59% for CCHFV, indicating a serious level of exposure [7,17]. To control and lessen this viral danger, a comprehensive strategy is required due to the rising risk of human infections and the underlying public health deficits. The first recorded incidence of CCHFV infection in Pakistan occurred in 1976 in a patient who had undergone laparotomy at Rawalpindi General Hospital. This led to a nosocomial outbreak that claimed three lives [67]. Eight nosocomial transmissions and fourteen epidemics were documented nationwide between 1976 and 2003, primarily in the northwest and western regions. Annual occurrences of CCHFV infection were documented from 1987 to 2001, with the first recorded case of CCHF occurring in Pakistan’s Baluchistan province in 1978. The majority of CCHF patients with verified immunoglobin M antibodies received their diagnoses between 2003 and 2008, according to the National Institute of Health in Islamabad [68]. The provinces of Baluchistan (57 cases), Khyber Pakhtunkhwa (20 cases), Punjab (6 cases), and Sindh (2 cases) had the highest number of confirmed cases. Additionally, from 2010 to 2014, 286 CCHF cases were documented, with a death rate of 20–29% [69]. The World Health Organization reported four deaths in the Khyber Pakhtunkhwa province in September 2013, two of which were linked to CCHF. In Abbottabad, Pakistan, all four victims were slaughterhouse employees and belonged to the same family [70]. Other CCHF cases reported in 2013 included two confirmed cases from the Killa Abdullah area and two suspected cases from the Musa Khail and Loralai districts in the province of Baluchistan. Of the 72 suspected cases, the majority were reported from various districts in Baluchistan, with a significant portion linked with same region where confirmed cases were identified. Consequently, the state reported 16 mortalities and 48 confirmed cases of CCHFV in 2013. Of the 62 suspected instances reported in 2012, 41 were confirmed cases, 18 of which resulted in fatalities [71]. Thirteen of these deaths were confirmed by laboratory testing, and five were suspected cases of CCHF. In addition, there were 23 confirmed cases from Baluchistan, 9 from Sindh, 6 from Khyber Pakhtunkhwa, and 5 from Punjab. This was a concerning CCHF outbreak in 2016, with 86 positive cases and a 41% death rate. Moreover, the majority of CCHF-positive patients were found between June and September of each year, with 55 cases recorded in 2017, 63 in 2018, 75 in 2019, and 28 in 2021. Between January and September of 2022, there were also 123 CCHF cases and 25 fatalities reported [72]. The Ministry of National Health Service, Regulations, and Coordination reported laboratory-confirmed CCHF cases from several regions of Pakistan, indicating a patchy distribution from everywhere [73,74]. A history of exposure, such as tick bites, contact with infected individuals, butchering or animal slaughter, handling or trading animals, handling animal hides, and employment in tanneries, was present in the majority of reported cases. A substantial amount of livestock and animal skins are traded between countries, including Pakistan, particularly with Iran and Afghanistan (Figure 1, Table 1) [5].

Pakistan has a high risk of contracting CCHFV due to several interrelated variables. One of the main problems is the lack of adequate medical facilities, especially in rural areas where medical infrastructure is frequently inadequate and unequipped to treat viral hemorrhagic fevers. Patient outcomes deteriorate as a result of this deficiency’s interference with prompt identification and therapy [75].

The situation is made worse by the notable lack of public understanding regarding tick control measures. Many people in endemic areas don’t know how to successfully lower their risk of tick exposure. To avoid tick bites, individuals should wear protective clothing, use insect repellents, avoid areas with dense vegetation, and check for ticks regularly after potential exposure, especially when there is a higher chance of human–animal contact [76]. In susceptible areas, a recurring cycle of illness and transmission is caused by the lack of effective educational efforts, which exacerbates the difficulties in vector management. To effectively tackle the public health hazard posed by CCHFV, a comprehensive strategy that prioritizes prevention, education, and healthcare improvement is necessary [77]. It is crucial to educate the public about the spread of CCHFV and prevent contact with potential infection sources. Educational campaigns should focus on providing practical guidelines on safe animal handling, personal protective measures, hygiene practices, and the risks of handling livestock, particularly during high-risk periods like the Eid-ul-Azha festival. The best way to implement these campaigns through media platforms, community outreach, and collaboration with local health authorities to ensure optimal efficacy and reach [76]. To keep an eye on tick populations and determine the risk of CCHFV transmission, it is essential to put in place continuous vector surveillance programs [77,78]. Involving local communities in public health programs, including forming cooperatives to manage ticks or conducting educational outreach, encourages a sense of responsibility and accountability for reducing the risks of CCHFV [72,79,80,81].

#### 2.2.3. Tamdy Virus (TAMV) and Karshi Virus (KSIV)

Arboviruses, which are mainly spread by arthropods, have drawn more attention as potential risks to public health because of their high rates of illness and mortality [82,83]. Karshi virus (KSIV) and Tamdy virus (TAMV) are two viruses that are still mostly unknown, especially in Pakistan. Although there have been prevalent reports of these viruses in neighboring countries, until recently, there was little information available about them in Pakistan [84,85]. This review highlights the findings of recent studies that reported their epidemiological pathways and potential public health interventions. Tick-borne viruses such as TAMV and KSIV are linked to livestock and the ectoparasites that feed on them. Their spread among tick populations and possible human spillover has been suggested by studies conducted in various regions. Nevertheless, there have been no verified human cases reported in Pakistan to date. Human serologic responses suggest that these viruses are silently circulating in this region, triggering the need for additional investigation into their ecology and transmission dynamics [86].

Tick species are the main vectors of both TAMV and KSIV transmission. It is thought that these viruses are amplified by livestock [87]. The ecological circumstances are conducive to spreading TAMV and KSIV because of the high frequency of tick infestations in Pakistan and the close interactions between animals and humans. The first concrete proof of TAMV and KSIV circulation in Pakistan was obtained by an epidemiological study in Faisalabad. The study found a KSIV seroprevalence of 1.10% (8/725; 95% CI: 0.56–2.16%) and a TAMV seroprevalence of 4.41% (32/725; 95% CI: 3.14–6.16%) using luciferase immunoprecipitation system (LIPS) tests. These results demonstrate that antibodies against these viruses are present in healthy people at a low but discernible prevalence [86]. Although these values point to possible exposure, it is crucial to remember that none of the samples presented TAMV or KSIV neutralizing activity, which raises concerns about the clinical significance and strength of immune responses against these viruses (Figure 1, Table 1) [86].

When TAMV- and KSIV-positive samples show minimal neutralizing activity, it may be a sign of either a weak immune response to these viruses or no active infection. The fact that antibodies have been found in a sizable section of the population despite this highlights the importance of being vigilant [86]. Tick-borne viruses, such as the Crimean–Congo hemorrhagic fever virus (CCHFV) and the severe fever with thrombocytopenia syndrome virus (SFTSV), are frequently linked to severe clinical syndromes [88]. The health risks associated with TAMV and KSIV may therefore be underestimated. Pakistan has little information about TAMV and KSIV because there are not any systematic surveillance or diagnostic tools established. Among the main difficulties are diagnostic gaps because current serological assays, such as LIPS, are helpful for preliminary detection but need to be validated by neutralization tests or other confirmatory techniques. To more accurately determine the prevalence and distribution of TAMV and KSIV, larger population-based studies are also desperately needed. Targeted entomological research is required since the significance of particular tick species in the spread of TAMV and KSIV in Pakistan is still unknown. Furthermore, the lack of documented human cases raises concerns about these viruses’ pathogenicity and clinical significance [86].

**Table 1:** Summarizes the reported cases of various Arboviruses in Pakistan, including the total reported cases, reported deaths, fatality rates of cases (%), host species, and vector.

Arbovirus	Year	Reported Cases	Reported Deaths	Case Fatality Rate %	Vectors	Host Species	References
Dengue Fever (DENV)	1994–2024	134985	360	0.26	*A. aegypti* & *A. albopictus*	Humans, Buffaloes, Rodents, Sheep, Cattle	[2,22,25]
Chikungunya Virus (CHIKV)	1983–2018	31771	-	-	*A. aegypti* & *A. albopictus*	Humans, Rodents	[30,31,32]
West Nile Virus (WNV)	1980–2024	398	1	0.25	*B. pipiens* & *C. quinquefasciatus*	Human, horses	[41,42,44]
Japanese Encephalitis (JE)	1980–2018	28	9	32.14	*C. tritaeniorhynchus.*	Humans	[49,50,51]
Zika Virus (ZIKV)	2021–2024	86	-	-	*A. aegypti* & *A. albopictus*	Humans, livestock	[53,55,86]
Severe Fever with Thrombocytopenia Syndrome (SFTSV)	2020–2024	900	-	-	-	Humans, livestock	[57,86]
Crimean-Congo Hemorrhagic Fever Virus (CCHFV)	1976–2024	1314	247	18.79	*H. anatolicum* *H. marginatum* *H. dromedarii* *Rhipicephalus* Spp.	Humans, Cattle, Sheep, goat	[72,77,86]
Tamdy Virus (TAMV)	2019–2014	32	-	-	-	Humans	[86]
Karshi Virus (KSIV)	2019–2014	8	-	-	-	Humans	[86]

## 3. Discussion

A serious public health concern is shown by the variety of arboviruses found in Pakistan and their reported seroprevalence. These diseases, which range from chikungunya and dengue to emerging viruses including CCHFV and SFTSV, are spread by mosquitoes and ticks. The exposure risks to these diseases are greatly affected by important environmental factors, including urbanization, climate change, and agricultural practices [7,57,58,89]. The growing geographic range of mosquito species in Pakistan is one major concern. Dengue was once limited to specific urban areas, but in recent years, the virus has moved to rural areas, possibly as a result of rising *A. aegypti* spurred by demographic shifts and urbanization. A similar example of the higher probability of arbovirus outbreaks in regions without efficient mosquito control techniques is the chikungunya virus, which was once isolated in the country and continues to spread [90,91].

Tick-borne diseases are spread via human–animal interactions as well as environmental factors. Increased human–livestock contact, particularly during seasonal celebrations like Eid-ul-Azha, is a direct cause of a rise in zoonotic illnesses like Crimean–Congo hemorrhagic fever (CCHFV) [92]. The intricacy of controlling vector-borne diseases in Pakistan is highlighted by the interaction of human behavior, tick exposure, and agricultural techniques [93,94]. The rising prevalence of numerous arbovirus co-exposure is a rising challenge. Co-infections, including concurrent dengue and chikungunya infections, make diagnosis and treatment more difficult because the symptoms of these diseases frequently overlap. Clinicians in these situations are forced to use advanced diagnostics, which are sometimes difficult to obtain in rural places. This emphasizes the necessity of rapid diagnostic testing as well as early warning systems to identify and distinguish between various arboviral diseases. Public health systems must be equipped with the necessary tools and training to deal with these challenges effectively [86]. Dengue has been the main focus of public education and awareness campaigns for arbovirus prevention, but other newly developing arboviral diseases including Zika, West Nile virus, and SFTSV also need to be addressed. In rural areas, where the hazards of diseases spread by ticks and mosquitoes are frequently underestimated, education programs should reach out beyond urban areas. A key component of these initiatives should be integrated vector management, which includes educating the public about avoid conditions that favor mosquitos’ life cycle and reducing livestock exposure to ticks. A significant constraint on this research is the absence of information regarding the frequency of arboviruses in Pakistani indigenous vectors and reservoirs. Although species like *A. aegypti*, *A. albopictus*, and ticks (*Hyalomma*, *Rhipicephalus*) are present, their role in arbovirus transmission remains unclear. Future research should concentrate on monitoring these vectors and reservoirs to determine the prevalence of the virus, as this is crucial for comprehending the dynamics of transmission and enhancing vector control strategies.

Finally, seroprevalence statistics from different parts of Pakistan suggest that a large number of people may have already been exposed to arboviral infections, with many asymptomatic cases remaining unreported. This emphasizes the significance of improving surveillance programs for tick and mosquito vectors and incorporating these initiatives into more comprehensive public health plans. In addition to controlling current epidemics, the healthcare system should promote a culture of anticipation and research to predict and prevent future outbreaks [86]. A comprehensive national health policy that emphasizes surveillance, early detection, and prevention must incorporate the current efforts to address arboviral infections in Pakistan. To reduce the risks and guarantee that Pakistan’s healthcare system is resilient to future arboviral threats, a comprehensive strategy that integrates public health policy, research, and community participation would be necessary.

## 4. Future Perspectives

Particularly in light of continuous climatic change, urbanization, and enduring socioeconomic difficulties, there is a considerable risk that diseases carried by ticks and mosquitoes would spread to new areas of Pakistan. Diseases like dengue, chikungunya, Zika, Crimean–Congo hemorrhagic fever (CCHFV), and tick-borne encephalitis are spread by vector species, such as *A. aegypti* and *A. albopictus* (mosquitoes) and *Hyalomma* and *Rhipicephalus* (ticks), whose geographic distribution has been altered by global warming. The potential of new outbreaks is increased as these vectors may spread into areas that were previously unaffected by these illnesses when temperatures rise and rainfall patterns change [95].

In addition to climate change, the population of mosquito-borne diseases is exacerbated by urbanization and rising population density in major cities such as Karachi, Lahore, and Islamabad, which create ideal circumstances for mosquito breeding [96]. At the same time, rural areas are more susceptible to tick-borne illnesses due to increased agricultural activities and close contact with livestock. Tick-borne infections are particularly likely to spread in areas with high livestock populations, notably in places like Punjab and Balochistan.

A multifaceted public health response is necessary to properly address these issues. Enhanced entomological monitoring, real-time disease surveillance systems, and upgraded virus and pathogen screening laboratories are vital components of a national strategy. This involves monitoring ticks in addition to mosquitoes to have a better understanding of the dynamics of diseases spread by ticks. To control mosquito and tick populations, priority should be given to integrated vector management (IVM), which incorporates insecticide-treated nets, indoor residual spraying, environmental management, and sustainable livestock tick control techniques [97].

Moreover, the deployment of a One Health approach which combines human, animal, and environmental health is crucial in addressing the interconnection of zoonotic diseases [98]. This strategy would encourage interdisciplinary cooperation among veterinarians, entomologists, environmental scientists, and medical professionals to address the underlying causes of disease transmission, including deforestation, poor sanitation, inappropriate waste disposal, and animal reservoir management [99]. Particular attention should be given to rural communities and livestock handlers who are particularly vulnerable to tick-borne diseases.

Response times during outbreaks will be greatly accelerated by raising public awareness of vector control methods and developing the ability of local public health authorities and healthcare providers. Reducing morbidity and mortality requires healthcare professionals to be trained in the rapid identification and diagnosis of diseases spread by ticks and mosquitoes. Communities in high-risk locations should also be the focus of public awareness campaigns that inform them of personal preventive measures, including applying insect repellent, using bed nets, removing breeding grounds, avoiding close contact with livestock, and getting medical help as soon as possible [98]. These efforts should also include preventive measures including treating livestock properly, avoiding ticks, and using acaricides in agricultural contexts. A comprehensive approach that simultaneously tackles diseases spread by ticks and mosquitoes will guarantee a more resilient and strong response to new zoonotic threats. This combined approach is critical in the face of climate change and environmental shifts that are altering the epidemiology of vector-borne diseases in Pakistan.

## 5. Conclusions

The epidemiology of diseases spread by mosquitoes and ticks in Pakistan is a challenging and dynamic public health issue that requires immediate awareness and control. The rise in diseases like dengue, chikungunya, and Crimean–Congo hemorrhagic fever become more severe due to urbanization, poor sanitation, climate change, and increased human–animal interactions. These factors work together to spread existing arboviral infections and to increase the risk of new pathogen outbreaks in previously unaffected areas.

There is a substantial silent burden of arboviral diseases, as shown by the evidence of broad seroprevalence rates among communities. This emphasizes the need for improved surveillance and monitoring methods. Implementing a One Health strategy that combines environmental, animal, and human health will promote interdisciplinary cooperation and provide a comprehensive framework for addressing the factors that contribute to the spread of zoonotic diseases.

To mitigate the spread of vector-borne diseases, a coordinated approach is essential. It is crucial to educate populations about vector-borne diseases hazards and protective measures. Educational programs should focus on enabling individuals to take preventive measures, such as wearing protective clothing, using insect repellent, and eliminating mosquito breeding grounds around their homes [46,47,60,80]. Advanced research facilities, surveillance, community education, vector control, appropriate waste management, and animal handling procedures should be the main focuses of disease control strategies. By integrating these strategies into a comprehensive public health framework, Pakistan can better address the challenges posed by vector-borne diseases and protect its population.

Furthermore, especially in high-risk locations, successful public health initiatives must place a strong priority on community education and awareness, emphasizing vector control strategies, appropriate waste management, and animal handling procedures. It is essential to establish advanced research facilities and strong real-time disease surveillance systems to enable prompt responses and lower the morbidity and fatality rates related to these diseases. A comprehensive approach that tackles the interconnected problems that arise from diseases spread by ticks and mosquitoes is ultimately necessary for Pakistan’s public health to thrive in the future. Pakistan might strengthen its ability to resist emerging arboviral threats and protect the health of its people in the face of urbanization and climate change by encouraging cross-sector cooperation, increasing community involvement, and funding research and infrastructure.

## Data Availability

All the data generated during the current study are included in the manuscript.
